# Impairment of T cell development and acute inflammatory response in HIV-1 Tat transgenic mice

**DOI:** 10.1038/srep13864

**Published:** 2015-09-07

**Authors:** Giuseppe Fiume, Annarita Scialdone, Francesco Albano, Annalisa Rossi, Franca Maria Tuccillo, Domenica Rea, Camillo Palmieri, Elisabetta Caiazzo, Carla Cicala, Claudio Bellevicine, Cristina Falcone, Eleonora Vecchio, Antonio Pisano, Simona Ceglia, Selena Mimmi, Enrico Iaccino, Annamaria de Laurentiis, Marilena Pontoriero, Valter Agosti, Giancarlo Troncone, Chiara Mignogna, Giuseppe Palma, Claudio Arra, Massimo Mallardo, Franco Maria Buonaguro, Giuseppe Scala, Ileana Quinto

**Affiliations:** 1 Department of Experimental and Clinical Medicine, University of Catanzaro “Magna Graecia”, Viale Europa, 88100, Catanzaro, Italy; 2 Molecular Biology and Viral Oncogenesis Unit, Department of Experimental Oncology, Istituto Nazionale Tumori “Fondazione Giovanni Pascale”, IRCCS, 80131, Naples, Italy; 3 Department of Pharmacy, University of Naples “Federico II”, Via Domenico Montesano 49, 80131, Naples, Italy; 4 Department of Public Health, University of Naples “Federico II”, Via Sergio Pansini 5, 80131, Naples, Italy; 5 Science of Health Department, University of Catanzaro “Magna Graecia”, Italy; 6 Department of Molecular Medicine and Medical Biotechnology, University of Naples “Federico II”, Via Sergio Pansini 5, 80131, Naples, Italy

## Abstract

Immune activation and chronic inflammation are hallmark features of HIV infection causing T-cell depletion and cellular immune dysfunction in AIDS. Here, we addressed the issue whether HIV-1 Tat could affect T cell development and acute inflammatory response by generating a transgenic mouse expressing Tat in lymphoid tissue. Tat-Tg mice showed thymus atrophy and the maturation block from DN4 to DP thymic subpopulations, resulting in CD4^+^ and CD8^+^ T cells depletion in peripheral blood. In Tat-positive thymus, we observed the increased p65/NF-κB activity and deregulated expression of cytokines/chemokines and microRNA-181a-1, which are involved in T-lymphopoiesis. Upon LPS intraperitoneal injection, Tat-Tg mice developed an abnormal acute inflammatory response, which was characterized by enhanced lethality and production of inflammatory cytokines. Based on these findings, Tat-Tg mouse could represent an animal model for testing adjunctive therapies of HIV-1-associated inflammation and immune deregulation.

The Human Immunodeficiency Virus type-1 (HIV-1), the ethiological agent of the acquired immune deficiency syndrome (AIDS), preferentially infects CD4^+^ cells of the immune system such as T-lymphocytes, monocytes, macrophages and dendritic cells, causing chronic immune activation and inflammation^1^,^2^. The HIV-1 Tat protein is essentially required for viral replication, and is a major pathogenic factor of AIDS-related immunological and neurological disorders^3^. As transcriptional transactivator, Tat binds to the 5′-untranslated RNA leader region of HIV-1, interleukin-6 (IL-6) and TNF-α, and enhances their expression through interaction with transcription factors, such as TBP, NF-κB and Sp1, and the histone acetyltransferases p300/CBP and P/CAF. Tat also recruits the cyclin T1/CDK9 complex to phosphorylate the carboxyl-terminus of the large subunit of RNA polymerase II, promoting elongation. Further, Tat affects cell signalling and metabolism by physical interaction with intracellular targets and membrane receptors, such as integrins, Flk1/KDR receptor and chemokine receptors^4^,^5^.

Tat plays a major role in inflammation and immune dysfunction in AIDS by altering the cytokine network^6^,^7^ and causing apoptosis of CD4^+^ T-cells^8^ and neurons^6^,^9^. In human monocytes and macrophages, Tat enhances the expression of pro-inflammatory cytokines, such as IL1-β, IL-6, IFN-α, IFN-γ, and TNF-α, and the immunosuppressive Th2 cytokine IL-10^10^,^11^,^12^,^13^, which indeed must be strictly and timely regulated to ensure the appropriate immune control of pathogens. In the gastrointestinal tract, HIV-1 infection causes immune hyper-activation, which results in the breakdown of mucosal barrier with the release of bacterial products in the blood to sustain chronic inflammation^2^. Tat also promotes severe microbial infections in AIDS by promoting the expression of co-infecting opportunistic pathogens, including Herpes simplex virus-1 (HSV-1), Kaposi’s sarcoma-associated herpesvirus (KSHV), Human Papilloma Virus (HPV), Mycobacterium avium and Mycobacterium tubercolosis, protozoa of Trypanosomatidae family, and Candida albicans^14^.

NF-κB transcription factors regulate the expression of genes involved in the immune response, inflammation and cell survival^15^. Inhibitors of NF-κB (IκBs) repress the NF-κB activity by associating with NF-κB dimers and impeding their binding to DNA^16^. Appropriate signalling causes the ubiquitination-coupled to proteasomal degradation of IκB repressors, releasing the NF-κB complexes for gene expression activation^17^. The NF-κB activity is strictly regulated to avoid the abnormal expression of NF-κB-dependent genes. Most cytokines involved in the development of immune system and immune control of pathogens are under the transcriptional control of NF-κB. NF-κB deregulation leads to immunological disorders, such as autoimmunity and immunodeficiency, as a consequence of altered expression of immunity functions. As mechanism of NF-κB deregulation in HIV-1 infection, we previously demonstrated that Tat up regulated the NF-κB activity through its physically association with the IκB-α inhibitor, impeding the repressor-mediated shut-off of NF-κB^18^. Moreover, Tat bound to the p65 subunit of NF-κB interfering the IκB-a repressor binding, and increasing the p65 recruitment to the NF-κB enhancer^18^. Further, in HIV-1-infected monocytes Tat enhanced the NF-κB-dependent expression of genes of immune response genes, including MIP-1α, CSF3, LTA, NFKBIA and TLR2^18^, which was consistent with similar findings in the sera of HIV-1 patients^19^,^20^. It is worthwhile to note that Tat presents some functional similarities with the HTLV-1-encoded Tax protein, which deregulates the NF-κB activity and affects cell proliferation, cell-cycle and apoptosis^21^,^22^,^23^.

Based on Tat-mediated deregulation of NF-κB, we addressed the question whether Tat could affect the development of lymphoid tissues and the acute inflammatory response in mice. To this end, we generated a transgenic mouse carrying the HIV-1 Tat gene under the control of the H2-K^b^ promoter and immunoglobulin heavy chain enhancer, which allowed to direct the Tat expression in lymphoid tissues.

## Results

### Impaired thymocytes differentiation in Tat-Tg mice

**Figure 1:**
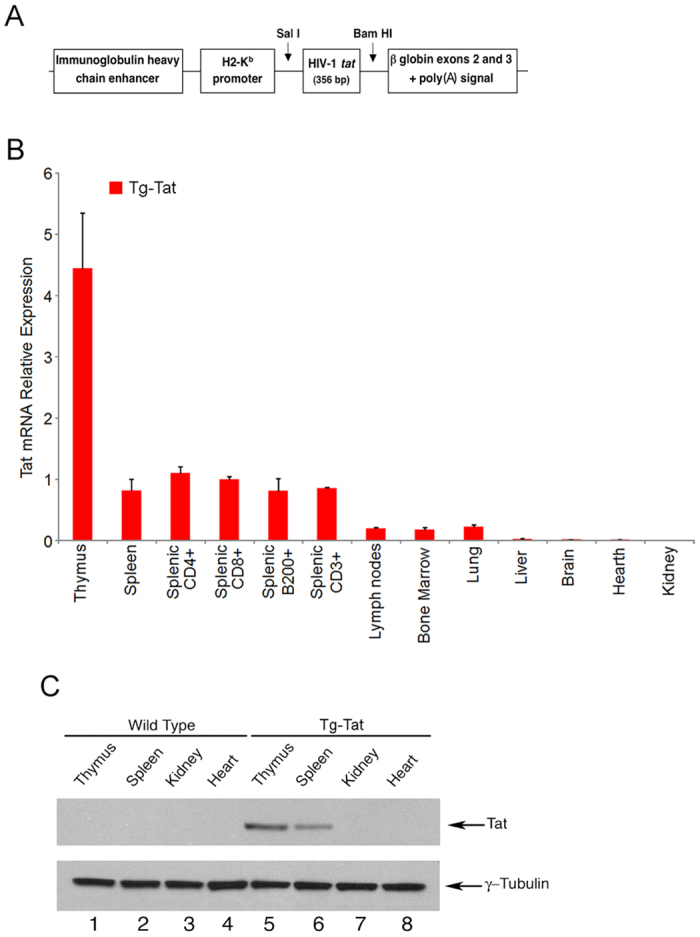
Expression of HIV-1 Tat in Tat-Tg mice. (**A**) Schematic representation of Tat-pHSE vector. (**B**) Expression of Tat mRNA in 8-weeks old Tat-Tg mice as measured by qRT-PCR of total RNA. Values (mean ± SE, n = 3) are shown. (**C**) Western blotting analysis of Tat in tissue extracts (40 μg) of 8-weeks old Tat-Tg mice. Data are representative of 3 independent experiments.

To analyse the effects of Tat on the immune system, we generated a transgenic mouse carrying the HIV-1 Tat 1–86 nucleotide sequence under the transcriptional control of the H2-K^b^ promoter and immunoglobulin heavy chain enhancer (Fig. 1A). As measured by qRT-PCR of total RNA, Tat was mainly expressed in the thymus and to a lesser extent in the spleen, lymph nodes, bone marrow and lung (Fig. 1B). In the spleen, Tat expression was observed in T-cells (CD3^+^ CD4^+^ and CD3^+^ CD8^+^) and B-cells (B220^+^). Differently, Tat was not expressed in the heart, kidney and liver (Fig. 1B). The expression of Tat protein in the thymus and spleen was confirmed by western blotting analysis (Fig. 1C). Tat was undetected in the serum of Tat-Tg mice by ELISA (Figure S1).

**Figure 2:**
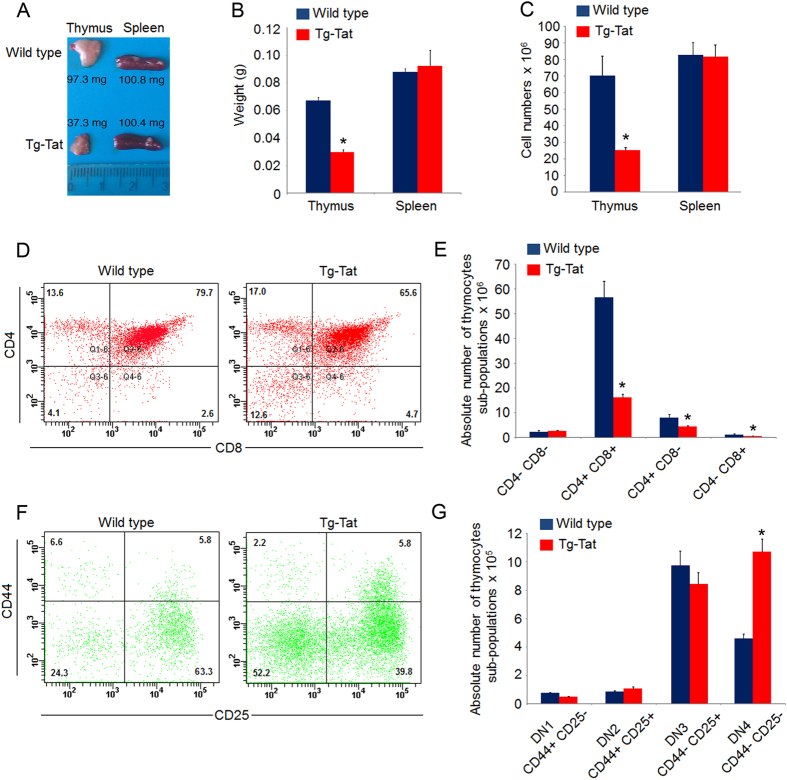
Aberrant thymopoiesis in Tat-Tg mice. (**A**) Thymus and spleen morphology of wild type and Tat-Tg mice. Scale bar is indicated. (**B**) Thymus and spleen weight of wild type and Tat-Tg mice. Values (mean ± SE, n = 6) are shown (**C**) Total thymocytes and splenocytes number of 8 weeks old wild type and Tat-Tg mice. Values (mean ± SE, n = 6) are shown. (**D**) Thymocytes (1 × 10^6^) of 8 weeks old wild type and Tat-Tg mice were stained with CD4-PE and CD8a-FITC. Each plot represents 20,000 events of a representative experiment with the percentage of thymic sub-populations. (**E**) Absolute number of thymic sub-populations of wild type and Tat-Tg mice. Values (mean ± SE, n = 6) are shown. The asterisk indicates a statistically significant difference among wild type and Tat-Tg mice, according to the Student’s t-test (*p* < 0.01). (**F**) Thymocytes (3 × 10^6^) of 8-weeks old wild type and Tat-Tg mice were stained with anti-CD3-FITC, CD4-FITC, CD8-FITC, CD25-APC, and CD44-PE antibodies. Triple negative (CD3^−^CD4^−^CD8^−^) population was gated on 500,000 events by FACS and analysed for the expression of CD44 and CD25. Percentages of thymic sub-populations are indicated. (**G**) Absolute numbers of DN1, DN2, DN3 and DN4 thymic sub-populations of wild type and Tat-Tg mice were calculated. Values (mean ± SE, n = 6) are shown. The asterisk indicates a statistically significant difference among wild type and Tat-Tg mice, according to the Student’s t-test (*p* < 0.01).

Tat-Tg mice were viable and fertile, and did not show any behaviour or gross anatomical alterations, except a significant reduction of thymus (size and weight) and total number of thymocytes (Fig. 2A–C). No differences were observed for the spleen (Fig. 2A–C). In Tat-Tg mice, the histological analysis showed the atrophy and altered architecture of thymus, including lack of lobular definition, cortex size reduction with faintly discernible cortical-medullary boundaries, and several cysts increasing with the animal age (Figure S2). Differently, the spleen architecture of Tat-Tg mice was unaffected (Figure S3).

T cell development occurs through sequential steps in the thymus, where CD4^−^CD8^−^ double negative (DN) cells progress toward CD4^+^ CD8^+^ double positive (DP) cells, which then differentiate toward CD4^+^ or CD8^+^ single positive (SP CD4 or SP CD8, respectively) T cells. In 8 weeks old Tat-Tg mice, we observed a percentage increase of DN cells (12.6% in Tat-Tg *vs* 4.1% in wild type), with decrease of DP cells (65.6% in Tat-Tg *vs* 79.7% in wild type), without significant differences of SP CD4 and SP CD8 cells (Fig. 2D). The absolute number of DP, SP CD4 and SP CD8 subpopulations was significantly reduced in Tat-Tg mice (Fig. 2E). Due to the increase of DN subpopulation, we investigated the sequential steps of DN maturation toward DP cells by FACS analysis of CD44 and CD25 expression in gated CD3- CD4- CD8- cells, according to the classification of DN1 (CD44^+^ CD25^−^), DN2 (CD44^+^ CD25^+^), DN3 (CD44^−^ CD25^+^) and DN4 (CD44^−^ CD25^−^) cells. Tat-Tg mice showed a percentage decrease of DN1 and DN3 subpopulations, and increase of DN4 cells (Fig. 2F), with a statistically significant augmentation of DN4 absolute number (Fig. 2G). In the spleen, no statistically significant difference was observed in percentage and absolute number of SP CD4, SP CD8 and B220+ B-cells sub-populations (Figure S4). These data indicated that Tat, even though expressed in both thymus and spleen, exclusively affected the thymus structure and thymocytes differentiation from DN4 to DP step.

### Deregulation of NF-κB activity and cytokines/chemokines expression in the thymus of Tat-Tg mice

**Figure 3:**
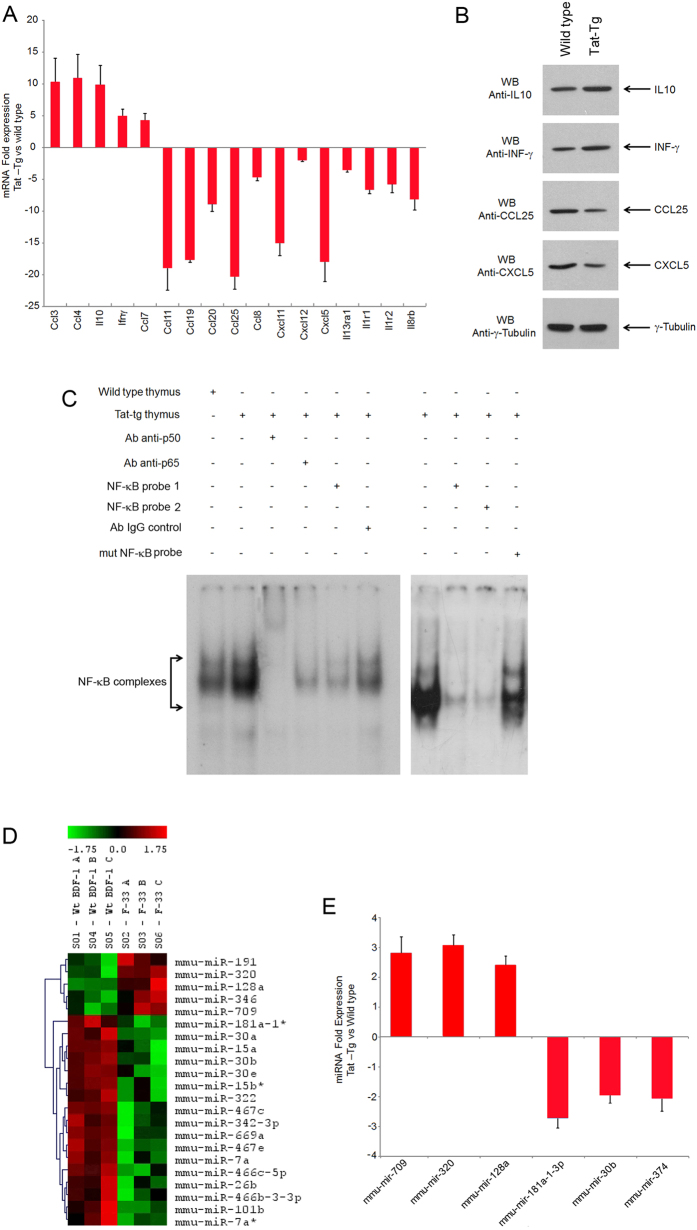
Altered expression of cytokines, chemokines, miRNAs and NF-κB activity in the thymus of Tat-Tg mice. (**A**) Total mRNA was extracted from thymus of 8 weeks old wild type and Tat-Tg mice, and analysed for the expression of inflammatory cytokines and chemokines by qRT-PCR using Mouse Cytokines & Chemokines Array (Qiagen). Values (mean ± SE, n = 3) are shown. (**B**) Western blotting analysis of cytokines and chemokines IL-10, INFG, CCL25, and CXCL5 in total protein extracts (20 μg) of thymus in wild type and Tat-Tg mice. (**C**) NF-κB DNA binding activity in thymocytes of wild type and Tat-Tg mice. Nuclear extracts (5 μg) were analysed for the DNA binding activity to the NF-κB double-stranded oligonucleotide by EMSA. For supershift, the samples were incubated with anti-p50 or anti-p65 antibodies. Cold competition was performed using wild type NF-κB double-stranded oligonucleotides (NF-κB probe 1, NF-κB probe 2) and a mutated NF-κB double-stranded oligonucleotide (mut NF-κB probe). (**D**) Heat map of miRNA expression in the thymus of 8 weeks old wild type and Tat-Tg mice. (**E**) Total mRNA (1 μg) from thymus of 8 weeks old wild type and Tat-Tg mice was analysed for expression of the indicated miRNAs by qRT-PCR. Fold induction of miRNA expression Tat-Tg vs wild type mice (mean ± SE, n = 3) is shown.

Thymocytes differentiation toward mature T lymphocytes is timely regulated by cytokines and chemokines that are produced at specific maturation steps^24^,^25^,^26^. Thus, we asked the question whether Tat affected the cytokines and chemokines expression in the thymus as mechanism of altered T-lymphopoiesis. The expression profile of 90 cytokines and chemokines was analysed by qRT-PCR of total RNAs extracted from thymus of 8 weeks old mice (Supplementary Table 1). Several cytokines and chemokines were up regulated (CCL3, CCL4, IL-10, INFG and CCL7), or down regulated (CCL11, CCL19, CCL20, CCL25, CCL8, CXCL11, CXCL12, CXCL5, IL13RA1, IL1R1, IL1R2, IL8RB) in Tat-Tg mice (Fig. 3A). The enhanced expression of IL-10 and INFG, and down-regulation of CCL25, and CXCL5 were confirmed at the protein level by western blotting analysis (Fig. 3B).

Consistently with the enhanced expression of inflammatory cytokines under NF-κB transcriptional control^18^,^27^,^28^, an increased p65 and p50 NF-κB DNA binding activity was observed in Tat-positive thymus as compared to wild type (Fig. 3C). These results suggested that the aberrant expression of cytokines/chemokines in the thymus of Tat-Tg mice likely affected the thymocytes differentiation.

### Altered microRNAs expression profile in the thymus of Tat mice

MicroRNA (miRNAs) regulate mRNAs expression at post-transcriptional level during thymus development and involution^29^,^30^,^31^. Tat was shown to modify miRNAs expression by acting as transcriptional transactivator of miRNA genes^32^, or as suppressor of RNA silencing (SRS) through inhibition of Dicer activity^33^. Thus, we analysed the genome-wide miRNAs expression profile in the thymus of wild type and Tat-Tg mice. A significant increase of 5 miRNAs and decrease of 17 miRNAs occurred in Tat-Tg mice (Fig. 3D). In particular, the up-regulation of microRNA-709, microRNA-320 and microRNA-128a, and down-regulation of microRNA-181a-1-3p, microRNA-30b and microRNA-374 were confirmed by RT-PCR (Fig. 3E). These results indicated that Tat modified the expression of specific miRNAs in the thymus, which could be relevant for T-lymphopoiesis.

### Peripheral blood lymphocytes and serum cytokines and chemokines in Tat-Tg mice

**Figure 4:**
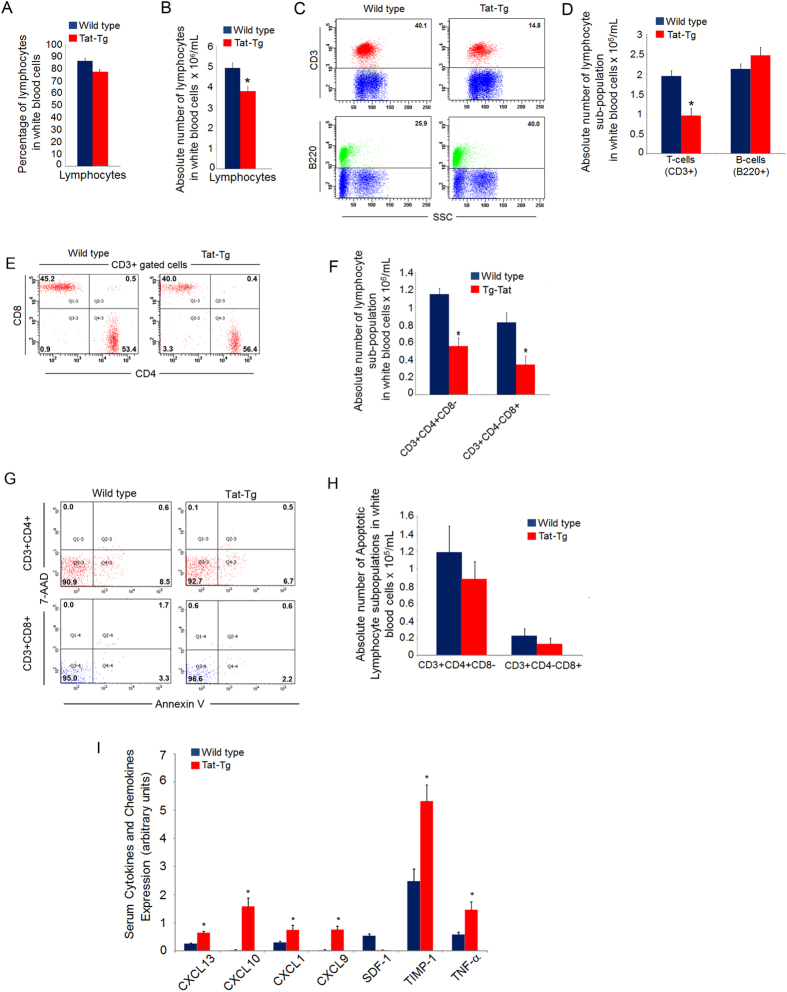
Depletion of CD4^+^ and CD8^+^ T-cells and altered cytokine/chemokines expression in peripheral blood of Tat-Tg mice. (**A**) Percentage and (**B**) absolute number of lymphocytes in peripheral blood of 8 weeks old wild type and Tat-Tg mice. Values (mean ± SE, n = 6) are shown. (**C**) Whole blood cells (1 × 10^6^) of 8 weeks old wild type and Tat-Tg mice were stained with anti-CD3-FITC and anti-B220-PE antibodies, and analysed by flow cytometry. Percentages of CD3^+^ T-lymphocytes (upper dot plots) and B220^+^ B-lymphocytes (bottom dot plots) of wild type and Tat-Tg mice are shown. Each plot represents 10,000 events of a representative experiment. (**D**) Absolute numbers of CD3^+^ T-lymphocytes and B220^+^ B-lymphocytes of wild type and Tat-Tg mice. Values (mean ± SE, n = 6) are shown. The asterisk indicates a statistically significant difference according to the Student’s *t* test (*p* < 0.01). (**E**) Whole blood cells (1 × 10^6^) of 8 weeks old wild type and Tat-Tg mice were stained with anti-CD3-APC, anti-CD45-V500, anti-CD14-V450, anti-CD4-PE and anti-CD8-FITC and analysed by flow cytometry. Percentages of CD3^+^ CD4^−^CD8^−^, CD3^+^CD4^+^CD8^+^, CD3^+^CD4^+^CD8^−^ and CD3^+^CD4^−^CD8^+^ of peripheral blood populations of wild type and Tat-Tg mice are shown. Each plot represents 20,000 events of a representative experiment. **(F)** Absolute number of CD3^+^ CD4^+^ CD8^−^ and CD3^+^ CD4^−^CD8^+^ cells. Values (mean ± SE, n = 6) are shown. The asterisk indicates a statistically significant difference according Student’s *t* test (p < 0.01). **(G)** Whole blood cells (1 × 10^6^) of 8 weeks old wild type and Tat-Tg mice were stained with anti-CD3-APC-Cy7, anti-CD4-PE, anti-CD8-FITC, Annexin V-APC, and 7-AAD and analysed by flow cytometry. Percentage of apoptotic CD3^+^ CD4^+^ and CD3^+^ CD8^+^ cells in wild type and Tat-Tg mice are shown. (**H**) Absolute number of apoptotic CD3^+^ CD4^+^ and CD3^+^ CD8^+^ cells in wild type and Tat-Tg mice are shown. Values (mean ± SE, n = 6) are shown. (**I**) Serum samples were obtained from retro-orbital blood of 8-weeks old wild type and Tat-Tg mice, and analysed for cytokines and chemokines expression, using the Mouse Cytokine Array Panel A (R&D System). Values (mean ± SE, n = 3) are shown. The asterisk indicates a statistically significant difference according Student’s *t* test (p < 0.01).

Next, we analysed the lymphocytes population in the peripheral blood of mice. By using an automated blood cell counter, a significant reduction of total lymphocytes was observed in Tat-Tg mice as compared to wild type (Fig. 4A,B). By FACS, we observed a significant decrease in percentage and absolute number of T-lymphocytes in the peripheral blood of Tat-Tg mice as compared to wild type (Figure S5A; Fig. 4C,D). A slight percentage increase of B cells was also observed in Tat-Tg mice without difference in absolute number (Fig. 4C,D). Moreover, by analysing the CD3^+^ T-lymphocyte sub-populations, the absolute number of CD3^+^ CD4^+^ and CD3^+^ CD8^+^ T cells were strongly reduced in Tat-Tg mice (Fig. 4E,F). Gated CD3^+^ cells were CD45^+^, and did not express the CD14 marker (Figure S5B,C), thus excluding the contamination with other cell subpopulations, such as monocytes. As apoptosis caused depletion of CD4^+^ and CD8^+^ T cells in HIV-1 infection^34^, we analysed apoptosis of CD3^+^ CD4^+^ and CD3^+^ CD8^+^ T cells by FACS. As measured by Annexin V/7-AAD assay, no statistically significant differences in percentage and absolute number of apoptotic CD3^+^ CD4^+^ and CD3^+^ CD8^+^ T-lymphocytes were observed in wild type and Tat-Tg mice (Fig. 4G,H). These findings suggest that Tat, affecting the late stage of T-lymphopoiesis, caused a reduction of circulating mature T cells. Further, 7 cytokines were differentially expressed in the serum of Tat-Tg mice, as measured by ELISA (Supplementary Table 2). In particular, CXCL13, CXCL10, CXCL1, CXCL9, TIMP-1 and TNF-α were up regulated, while CXCL12 was down regulated (Fig. 4I). These results demonstrated an increased level of circulating inflammatory cytokines in Tat-Tg mice as hallmark of chronic inflammation.

As chronic inflammation in HIV-1 infection determined the breakdown of mucosal barrier^2^, we addressed the question whether Tat affected the integrity of intestinal epithelium. Histological analysis of large bowel sections showed a normal glandular epithelium without intra-epithelial lymphocytic infiltrates in wild type and Tat-Tg mice (Figure S6A). By immunohistochemical analysis, a few perivascular CD4^+^ T lymphocytes were detected in both animal groups in absence of a significant intra-epithelial component (Figure S6A). We also observed a strong reactivity to anti-Occludin, which is a tight junction marker, thus indicating that the integrity of intestinal epithelium was unaffected in wild type and Tat-Tg mice (Figure S6A). By TUNEL assay, there was no significant enterocytes apoptosis in the same large bowel sections of wild type and Tat-Tg mice (Figure S6B). Altogether these results exclude a role of Tat in the breakdown of mucosal barrier.

### Abnormal inflammatory response to LPS in Tat-Tg mice

**Figure 5:**
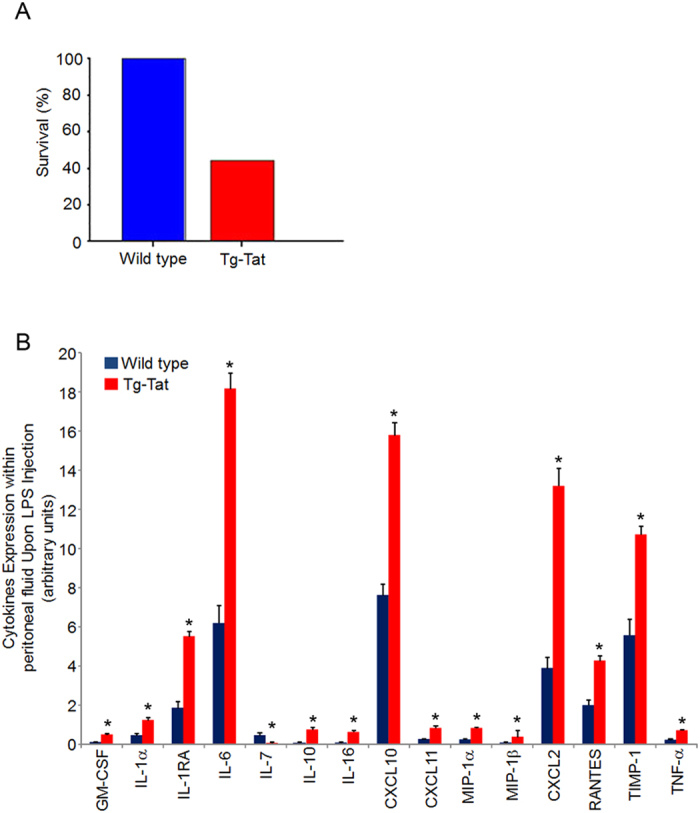
Enhanced acute inflammatory response to LPS in Tat-Tg mice. *E. coli* LPS (serotype 0127:B8) (15 mg/kg) was i.p. injected in 8-weeks old wild type and Tat-Tg mice. (**A**) Survival rates of mice 24 hrs after LPS injection. (**B**) Intra-peritoneal lavages were collected and analysed for cytokines and chemokines protein expression. Values (mean ± SE, n = 3) are shown. The asterisk indicates a statistically significant difference according Student’s *t* test (p < 0.01).

Next, we asked the question whether the increased production of inflammatory cytokines together with mild reduction of circulating T cells could affect the acute inflammatory response in Tat-Tg mice. Thus, we challenged mice with lipopolysaccharide (LPS) endotoxin, which is component of gram-negative bacterial wall causing septic shock^35^,^36^,^37^. Wild type and Tat-Tg mice were intra-peritoneally injected with LPS (15 mg/Kg) and monitored for survival. At 24 hours post-injection, 40% of Tat-Tg mice survived as compared to 100% of wild type (Fig. 5A). In peritoneal lavage fluids of survived Tat-Tg mice, we observed a statistically significant increase of GM-CSF, IL-1α, IL-1RA, IL-6, IL-10, IL16, CXCL10, CXCL11, MIP-1α, MIP-1β, CXCL2, RANTES, TIMP-1 and TNF-α, and decrease of IL-7 as compared to wild type (Fig. 5B; Supplementary Table 3). Altogether these results indicated that Tat-Tg mice were more susceptible to LPS-induced death, and had a higher production of inflammatory cytokines, including IL-1α and TNF-α, which are key factors in the development of septic disease.

## Discussion

Tat is an essential protein for HIV-1 replication and AIDS pathogenesis. Besides its role as transcriptional activator of HIV-1 and cellular genes, Tat affects the cellular metabolism through physical interaction with different cellular targets. Mostly relevant for the immune system, Tat counteracts the action of the IκB-α repressor of NF-κB and increases the transcriptional activity of the p65 subunit of NF-κB, thus enhancing the NF-κB activity and the expression of inflammatory cytokines and chemokines^18^. As additional mechanism of inflammation, Tat promotes the expression of IL-6 and TNF-α gene through the binding to their 5′ untranslated leader mRNAs^13^,^38^.

NF-κB activates the expression of genes involved in the development of immune system and immune response, and is timely regulated by the immune signalling^15^. Deregulation of the NF-κB activity can alter the immune defence against pathogens, and eventually cause immunodeficiency or autoimmunity^15^. Based on the constitutive activation of NF-κB by Tat^18^, we addressed the question whether Tat could affect T-lymphopoiesis and alter the acute inflammatory response to LPS. To this end, we generated a transgenic mouse that expressed HIV-1 Tat under the H2-K^b^ promoter and immunoglobulin heavy chain enhancer to allow the specific expression of Tat in lymphoid tissues^39^,^40^,^41^. In Tat-Tg mice, Tat was peculiarly expressed in the thymus and to a lesser extent in the spleen, lymph nodes, bone marrow and lung. Thymus atrophy and thymocytes depletion was observed in Tat-Tg mice with 3D structural alterations of thymic architecture, including cystic formations arising at earlier time as compared to wild type (Figure S2). Crosstalk signals deriving from developing thymocytes are relevant for appropriate 3D configuration of thymus structure. In fact, depletion of thymic precursors was associated with cystic involution of thymus^42^. In this regard, the early appearance of cystic formations in Tat-Tg mice could be a sign of thymus involution due to alteration of thymocytes compartments. Consistently, Tat-Tg mice had an impaired thymopoiesis with increased percentage of DN (CD4^-^ CD8^-^) cells, and decrease of DP (CD4^+^ CD8^+^), SP (CD4^+^ CD8^-^) and SP (CD4^-^ CD8^+^) cells. In particular, we observed an increased DN4 subpopulation of DN compartment of Tat-Tg mice, suggesting a partial maturation block from DN4 toward DP differentiation step. These findings are consistent with a defective thymopoiesis and thymus destruction occurring in HIV-1 infection as consequence of increased susceptibility of human thymocytes and thymic stromal cells to the virus^43^. As unique source of CD4^+^ and CD8^+^ T cells deriving from the sequential maturation steps of DN and DP cells, the thymus is essential for T-cell renewal, and is a major target of HIV-1 infection^44^. In adults, the importance of a functional thymus is not fully understood. However, the thymus is considered to be critical in HIV disease, in which T cells are depleted in large numbers. Alterations of thymic structure and function were previously reported in HIV-1 infection^45^; in particular, histopathological and morphological evidence of thymic dysfunction occurred in infants with rapid onset of severe AIDS^46^. HIV-1 and simian immunodeficiency virus (SIV) can infect thymocytes *in vivo* and *in vitro*^47^,^48^, causing thymic pathological conditions. In humans, the thymic atrophy is a recurrent finding characterized by an increased number of DN thymocytes and depletion of DP and SP (CD4^+^ CD8^-^) thymocytes^46^,^49^. In HIV-1-infected children, loss of peripheral CD4^+^ and CD8^+^ T cells derives from a severe loss of DP cells^50^; similar findings were observed in SIV-infected macaques^51^,^52^. Thus, in Tat-Tg mice, the thymic atrophy associated with increased DN population and decreased DP population resembles the alterations occurring in HIV-1/SIV infection, especially in paediatric AIDS, suggesting that Tat is a major viral protein involved in aberrant thymopoiesis in AIDS.

Tat-Tg mice did not develop tumours up to 18 months observation. Differently, other transgenic mice that ubiquitously expressed Tat under the SV40 promoter developed dermal lesions resembling Kaposi’s sarcoma^53^,^54^, which suggests a potential oncogenic role of Tat depending on tissue-specific expression. Moreover, Tax-transgenic mice, where the HTLV-Tax gene expression was under the Lck promoter and restricted to thymocytes, developed T-cell leukemia-lymphoma^55^. In this regard, the lack of oncogenic activity of Tat in Tat-Tg mice here described remarks a different action of Tat as compared to Tax, as Tat mainly affects T-lymphopoiesis.

NF-κB activity must be strictly regulated in the course of thymocytes differentiation with activity oscillations at specific maturation steps^15^. In DN compartment, a low NF-κB activity is usually observed at DN1 and DN2 stages, followed by an increase at DN3 stage, and rapid decrease in the DN4 transition toward DP cells^15^. Consistently with Tat-dependent up-regulation of NF-κB^18^, the p65/NF-κB DNA binding activity and expression of NF-κB-dependent MIP-1α, MIP-1β and INFγ genes were increased in the thymus of Tat-Tg mice. These findings suggest that the Tat-dependent up-regulation of NF-κB likely interfered the DN4 differentiation toward DP cells, as the NF-κB activity must be down regulated at this specific step of thymocytes differentiation^15^. As additional effect, Tat lowered the thymic expression of CXCL12 and CCL25, which are required for thymocytes maturation from DN3/DN4 to DP stage^24^,^25^,^26^. The mechanism of CXCL12 and CCL25 down-regulation could likely involve the Tat interaction with cellular functions that are involved in the CXCL12/CCL25 gene expression control.

MiRNAs regulate the gene expression at post-transcriptional level during immune system development^30^,^31^. Consistently with previous reports^56^,^57^,^58^, we found a number of differentially expressed miRNAs in the thymus of Tat-Tg mice as compared to wild type. Among others, microRNA-181a-1-3p was significantly down regulated in Tat-Tg mice. Over-expression of the precursor mir-181a-1 in DN thymic progenitors was previously shown to increase the number of DP cells^59^. In this regard, the evidence of microRNA-181a-1-3p down-regulation associated with DP cells reduction in the thymus of Tat-Tg mice suggests that the partial maturation block from DN4 to DP step could be consequence of Tat repression of mir-181a-1-3p expression. In peripheral blood, CD4^+^ and CD8^+^T lymphocytes were significantly reduced in Tat-Tg mice. These findings could be due to the low production of mature SP CD4^+^ and SP CD8^+^ cells in the thymus of Tat-Tg mice, and are consistent with the depletion of peripheral CD4^+^ and CD8^+^ T cells in HIV-1-infected children^50^. Apoptosis caused the depletion of CD4^+^ and CD8^+^ T cells in HIV-1 infection^34^. In Tat-Tg mice, any increase in CD4^+^ and CD8^+^ T cells apoptosis was observed in peripheral blood, thus supporting the hypothesis that Tat caused a depletion of circulating T cells by mainly deregulating T-lymphopoiesis. Tat-Tg mice also showed an altered expression profile of serum cytokines and chemokines, which resembled the one reported in progressive AIDS^19^,^60^. In particular, we observed the increase of IL-1α IL-1Rα, TNF-α, IL-6 and CXCL10, which arise soon after HIV-1 detection and acute infection^19^,^60^, and the decrease of IL-10, which is peculiar of late stage of HIV-1 infection^19^,^60^.

Immune dysfunction and immunosuppression are hallmark features of septic syndrome and a major cause of mortality. In particular, HIV-1 patients show a higher susceptibility to septic shock as consequence of severe course of opportunistic diseases, immunodeficiency and persistent inflammation^14^. In this regard, Tat-Tg mice were more susceptible to the systemic inflammatory response syndrome caused by the intra-peritoneal injection of LPS, based on the evidence of increased lethality and expression levels of TNF-α, IL-1α, IL-6 and CXCL10 in the peritoneal lavages of surviving animals. These findings were consistent with the typical extreme “cytokines storm” characterized by chronically elevated or excessive production of inflammatory cytokines, including TNF-α, IL-1α, IL-6 and CXCL10^10^. Chronic inflammation in HIV-1 infection is an emerging cause of non-AIDS comorbidities, such as cardiovascular and cerebrovascular disease, diabetes, chronic kidney disease, osteoporosis and cancer, in patients treated with antiretroviral therapy^61^. In this perspective, the Tat transgenic mouse could represent an animal model of AIDS-related chronic inflammation and immune dysfunction, in order to test novel therapeutic strategies to counteract the Tat-dependent immune deregulation.

## Methods

### Plasmids

The plasmid pHSE-Tat was generated by cloning the HIV-1 Tat (1–86) cDNA of HTLVIIIB strain in the BamHI/SalI restriction sites of pHSE under the H-2K^b^ promoter and the immunoglobulin heavy chain enhancer, which direct the expression of Tat in lymphoid tissues^39^,^40^,^41^.

### Generation of Tat transgenic mouse

The Tat transgenic mouse was generated according to established procedures^62^,^63^,^64^. Briefly, the pHSE-Tat plasmid was linearized by cutting the XhoI restriction site, and microinjected (200 ng) into the pro-nuclei of fertilized eggs from super-ovulated B6D2F1/J female mice (Charles River Laboratories, Italy). B6D2F1/J mice are a hybrid strain generated by crossing C57BL/6 females with DBA/2 males. Genomic DNA extracted from offspring tails was analysed by dot blot hybridization with the ^32^P-labelled Tat cDNA for the presence of Tat gene. A founder mouse was mated with normal B6D2F1/J mice, and offspring was backcrossed for 8 generations. Heterozygous Tat transgenic mice were inter-crossed to obtain homozygous Tat mice. Animals were maintained in a dedicated pathogen-free animal facility. Mice experiments were performed with the approval of the ethical committee, as detailed in the Ethical Statement section. For genotyping, DNA was extracted from mice tails by using Extract-N-Amp Tissue PCR kit (Sigma-Aldrich). The presence of Tat gene in the genomic DNA was verified by PCR using the following primers: 5′Tat: 5′- TTACTCGACAGAGGAGAGCAAGAA-3′ and 3′Tat: 5′- TCTCTGTCTCTCTCTCCACCTTCTTCTTCTAT-3′. PCR amplification was performed using Taq DNA Polymerase (Invitrogen, Carlsbad, USA) under the following conditions: 94 °C, 2 min; (94 °C, 15 s; 58 °C, 30 s; 72 °C, 15 s) × 30 cycles; 72 °C, 10 min.

### Real-Time PCR

Total RNA was extracted from homogenized tissues with the TRIzol reagent (Invitrogen) and treated with DNase I AmpliGrade (Life Technologies, Italy), as previously described^65^,^66^,^67^. RNA aliquots (200 ng) were reverse transcribed using Random Examers (Roche Diagnostic GmbH, Mannheim, Germany) and Superscript III Reverse Transcriptase (Invitrogen), according to the manufacturer’s protocol. Real-time PCR was performed with the iQ Green Super mix (Bio-Rad Laboratories, Hercules, CA, USA) and carried out with the iCycler iQ Real-Time detection system (Bio-Rad Laboratories) under the following conditions: 95°C, 1 min; (94°C, 10 s; 60°C, 30  s) × 40 cycles. The primers used to detect Tat expression were: forward 5′- CCCTGGAAGCATCCAGGAA-3′; reverse 5′-TCTTCGTCGCTGTCTCCGCT-3′. To measure the cytokines and chemokines expression, RT-PCR was performed using the Mouse Cytokines & Chemokines RT^2^ Profiler PCR Array profiles (QIAGEN Sciences, MD, USA). Reactions were carried out in triplicate, and gene expression levels were calculated relative to HPRT1 mRNA levels as endogenous control. Relative expression was calculated as 2(Ct ^gene under investigation^ − Ct ^HPRT1^), as previously described^68^,^69^,^70^,^71^,^72^. MiRNAs expression was measured by Taqman assays using TaqMan Universal PCR Master Mix, No AmpErase UNG (Life Technologies), according to manufacturer’s conditions. The following primers (Life Technologies) were used: miR-709 (RT1644 and TM1644), miR320 (RT536, TM536), miR-128a (RT453, TM453), miR-181-a1* (RT516, TM516), miR-30b (RT602, TM602), miR-374-5p (RT1319, TM1319). Sno-234 (RT1234, TM1234) and sno-429 (RT1240, TM1240) were used as internal normalizer.

### Cells extracts, Western blotting and NF-κB DNA binding

Total cell extracts were performed as previously described^73^,^74^. Briefly, cells were isolated from different organs, washed twice with PBS 1X and lysed in RIPA buffer, containing 1% NP-40, 10 mM Tris-HCl, 150 mM NaCl and 1 mM EDTA, supplemented with a protease inhibitor cocktail (cOmplete-mini EDTA-free tablets – Roche) on ice for 20 min. Lysates were clarified by 10 min centrifugation at 14,000 × *g*, 4 °C. Western blotting was performed by resuspending protein aliquots in loading buffer (125 mM Tris–HCl, pH 6.8, 5% SDS, 1% bromophenol blue, 10% β-mercaptoethanol, 25% glycerol), resolved on 12% SDS–PAGE, transferred to polyvinylidene difluoride membrane (Millipore, Bedford, MA, USA) and incubated with primary antibodies (1:1000) followed by incubation with horseradish-peroxidase-linked mouse or rabbit IgG (1:2000) (GE Healthcare Amersham, Little Chalfont, UK) in PBS containing 5% non-fat dry milk (Bio-Rad Laboratories). Proteins were detected by chemiluminescence using the ECL System (GE Healthcare Amersham). Primary antibodies were purchased from Sigma-Aldrich (anti-γ-Tubulin) or Santa Cruz Biotechnology (anti-IL-10, sc-73309; anti-IFNG, sc-59992; anti-TECK/CCL25, sc80344; anti-CXCL5, sc-103487) while Tat protein was detected by using HIV-1 Tat mAb (5A5.3) mAb (1 μg/mL, AIDS Research Reagent Program, Division of AIDS, NIAID, NIH), followed by incubation with a anti-mouse-HRP (GE Healthcare). Nuclear extracts were performed as previously described^75^. Briefly, thymocytes were isolated from freshly-extracted thymus of wild-type and Tat-Tg mice, dissociated in RPMI using a syringe and forceps, washed twice in PBS and resuspended in lysis buffer containing 10 mM N-2- hydroxyethylpiperazine-N’-2 sulfonic acid (HEPES) pH 7.9, 1.5 mM MgCl2, 10 mM KCl, 0.5 mM dithiotreithol (DTT) and 0.1% NP-40. Cells were lysed on ice for 2 min and checked for complete lysis by microscopy. Nuclei were centrifuged at 800 × *g* for 5 min and supernatant was collected as cytosolic extract. The nuclear pellet was washed with a buffer containing 10 mM HEPES pH 7.9, 1.5 mM MgCl2, 10 mM KCl, 0.5 mM DTT and lysed in a buffer containing 20 mM HEPES pH 7.9, 25% glycerol, 0.45 M NaCl, 1.5 mM MgCl2, 0.2 mM EDTA, 0.5 mM DTT, 1 mM PMSF, 1× Complete Protease Inhibitor; nuclear lysates were clarified by centrifugation at 14,000 × *g* for 15 min and supernatant was collected as nuclear extract.

For NF-κB DNA binding analysis, nuclear extracts were collected and Electrophoretic Mobility Shift Assay (EMSA) was performed, as previously described^75^. Briefly, NF-κB oligonucleotide probe 1 (Promega, cat. E3291, 5′-AGT TGA GGG GAC TTT CCC AGG C-3′) was end-labelled with [γ-32P]ATP (Perkin Elmer) using polynucleotide kinase (Lifetech). Equal amounts (5 μg) of nuclear extracts were incubated in a 20 μl reaction mixture containing 10% glycerol, 60 mM KCl, 1 mM EDTA, 1 mM DTT, and 2 μg of poly [d(G-C)] (Boehringer Mannheim, Germany), in presence or absence of anti-p65, anti-p50 or IgG isotype control antibodies. One μl of [γ^32^P]-labelled double-stranded probe (0.2 ng, 5 × 10^4 ^cpm) was then added in presence or absence of 10-fold molar excess of cold competitor oligonucleotide. Cold competitor probes were: NF-κB probe 1 (5′-AGT TGA GGG GAC TTT CCC AGG C-3′), NF-κB probe 2 (HIV-1 NF-κB enhancer, 5′-ATCAGGGACTTTCCGCTGGGGACTTTCCG-3′), and a mutated NF-κB probe (5′- AGT TGA GG**C** GAC TTT CCC AGG C -3′, where the mutated base is indicated in bold). The reactions were incubated at room temperature for 15 min, and run on a 6% acrylamide:bisacrylamide (30:1) gel in 22.5 mM Tris borate, 0.5 mM EDTA. Gels were dried and autoradiographed.

### ELISA

Ninety-six wells Maxisorp microtiter plates (NUNC) were coated with 100 μL/well containing 30 μg of serum or thymic protein extracts of wild type or Tat-Tg mice, and incubated over-night at 4°C. The following day, the plate was washed and blocked in 200 μL of blocking buffer (PBS 1 × , 5% non-fat dry milk, 0.01% tween 20) and next incubated in the same buffer in presence or absence of anti-Tat antibody (HIV-1 Tat mAb 5A5.3) (1 μg/mL) for 2 h at 37 °C. Next, each well was washed six times with PBS 1 × containing 0.05% tween 20, and then incubated with a 1:5000 dilution of Alkalyne Phosphatase conjugated goat anti-mouse IgG for 1 h at 37 °C. After extensive washing, samples were incubated with PNPP (Para-Nitro-Phenyl-Phosphate) platelets substrate, diluted in Diethanolamine substrate solution pH 9.6, for 10 min at room temperature. Optical density (OD) was measured twice at 420 nm on a MULTISCAN EX ELISA Plate reader (Thermo Labsystems, Franklin, MA).

### MiRNA microarray

Total RNA was extracted from thymus of 8 weeks old wild type and Tat-Tg mice (3 mice per group) with Trizol reagent and miRNA microarray was performed by LC Sciences Service (Houston, TX, USA; http://www.lcsciences.com/) using μParaFlo microfluidic chip technology (LC Sciences) with probes for miRNAs listed in Sanger miRBase release 10.0. The Accession number of this experiment at GEO Dataset is GSE65006.

### Flow cytometry

For flow cytometry, the following antibodies were purchased from BD Biosciences: CD25-APC (PC61), CD3-FITC (17A2), CD3-APC-Cy7, CD4-PE (L3T4), CD8a-FITC (LY-2, 53-6.7), CD8a-APC (53-6.7), B220-PE (CD45RA-14.8), Annexin V-APC, CD45-V500, CD14-V450, IgG_2a_-PE rat isotype (R35–95), IgG_2a_-APC rat isotype), IgG_2a_-FITC, IgG_2a_-V500, IgG_2a_-V450 rat isotype. CD44-PE (IM7) was purchased from Biolegend, San Diego, CA, USA. Thymocytes, splenocytes and whole blood cells (1 × 10^6^ cells) were incubated with fluorochrome-conjugated antibodies (0.5 μg) for 30 minutes on ice, avoiding light. After two PBS washes, cells were analysed by flow cytometry using BD FACSAria (BD Biosciences) flow cytometer and BD FACSDiva software (BD Biosciences), as previously described^76^. Splenic B-cells (B220+), splenic T-cells (CD3+), splenic CD4+ and CD8+ cells were sorted by antigenic criteria using BD FACSAria flow cytometer/cell sorter, with a purity grade of 99%.

### Differential blood cell count

Blood samples were collected from retro-orbital plexus of 8 weeks old mice by capillarity in a tube containing EDTA 0.5M. Aliquots of blood samples (100 μL) were analysed by using MS4–5 Veterinary Haematology analyser (MELET SCHLOESING Laboratories, France).

### Cytokines and chemokines expression

Serum samples were obtained from blood samples of 8 weeks old mice, allowed to clot for 30 min at room temperature, and then centrifuged at 3000 × *g* for 15 min. Serum aliquots (150 μL) were immediately assayed for cytokines and chemokines expression by using Mouse Cytokine Array Panel A (ARY006) (R&D System, Minneapolis, USA), according the manufacturer’s conditions. Cytokines/chemokines expression levels were calculated as arbitrary units by acquiring images of developed X-ray film of cytokine array with an Image Scanner (EPSON), and measuring pixel densities of the spots with ImageJ software (ImageJ, USA).

### LPS challange

B6D2F1 wild type mice (4 females and 4 males) and Tat-Tg mice (5 females and 4 males) were intra-peritoneally injected with LPS (*E. coli, serotype 0127:B8*, Sigma) (15mg/Kg of body weight). Twenty-four hours post-injection survival rates were evaluated and surviving mice underwent to peritoneal lavages performed with 2 ml of heparinized (10 U/ml) Dulbecco’s Modified Eagle Medium (DMEM). Peritoneal fluids were centrifuged at 2500 × *g* for 10 min, and the expression of cytokines and chemokines was measured in collected supernatants using Mouse Cytokine Array Panel A (R&D System, ARY006), according the manufacturer’s protocol.

### Histology, Immunohistochemistry and Fluorescence Microscopy

Thymus, spleen or large bowel of mice were wholly removed at necropsy, fixed in buffered formalin (10%) dehydrated and paraffin embedded. Thick sections (5 μm) of each paraffin block were stained by hematoxylin and eosin or used for immunohistochemistry or fluorescent microscopy. For immunohistochemical analysis, slides were deparaffinized, rehydrated and immersed in 10 mM citric acid, pH 6, in a microwave oven (VWR International PBI Srl, Milan, Italy) to exclude epitope masking owing to fixation. Sections were immunostained with primary antibodies against CD4 and Occludin and detected with an indirect immunoperoxidase technique employing UltraVision LP Detection System HRP Polymer & DAB Plus Chromogen (Thermo Fisher Scientific Inc., Waltham, MA, USA), according to the manufacturer’s protocol. As negative controls, sections were incubated with PBS solution (Bio-Optica Milano SpA). Nuclear staining was carried out using hematoxylin. Microscopic analysis was performed with a Leica DMLB microscope (Leica Microsystems, Wetzlar, Germany). Images were taken in bright field with a digital camera (Leica DC200; Leica Microsystems) connected to the microscope. For fluorescence microscopy, slides were stained using *In Situ* Cell Death Detection Kit, TMR red (Roche Diagnostics), following manufacturer’s instructions. Mounting was performed using Vectashield Antifade Mounting Medium with DAPI (Vector laboratories). Signals were visualized with a Leica DMLB fluorescence microscope. Images were processed as one-color images, merged and reconstructed using Adobe Photoshop software.

### Statistical analysis

Statistical analysis was performed by the two-tailed unpaired Student’s *t* test. Data were reported as mean values ± SE. Differences between the means were accepted as statistically significant at the 99% level (*p* < 0.01).

### Ethics Statements

Mice were housed at the Animal Facility of Istituto Nazionale Tumori IRCCS Fondazione “G. Pascale”. Experiments were carried out in conformity with protocols (N. 657–2011 entitled “Phenotypic and immunological characterization of HIV-1 Tat-transgenic mice” and N. 959–2013 entitled “Phenotypic analysis of Tat-transgenic mice as a model of inflammation”) approved by the Veterinary Department of the Italian Ministry of Health, in accordance to the ethical and safety rules and guidelines for the use of animals in biomedical research provided by the relevant Italian laws, indicated in *art. 4–5 of D.lgs 116/92, DD.MM. of 29/09/1995* and *26/04/2000*, and in accordance with the ethical guidelines for animal care of the European Community Council (*directive n. 86/609/ECC*). All efforts were made to minimize the animal’s suffering.

## Additional Information

**How to cite this article**: Fiume, G. *et al.* Impairment of T cell development and acute inflammatory response in HIV-1 Tat transgenic mice. *Sci. Rep.* **5**, 13864; doi: 10.1038/srep13864 (2015).

## Supplementary Material

Supplementary Information
