# Supplementary material for: Impairment of T cell development and acute inflammatory response in HIV-1 Tat transgenic mice
Source: Sci Rep. 2015 Sep 7;5:13864. doi: 10.1038/srep13864 (PMC4561375; doi:10.1038/srep13864)
Supplement: Supplementary Information [file srep13864-s1.pdf]

Supplementary Materials

Impairment of T cell development and acute inflammatory response in HIV-1 Tat transgenic mice

Giuseppe Fiume[1,*], Annarita Scialdone[1], Francesco Albano[1], Annalisa Rossi[1], Franca Maria Tuccillo[2], Domenica Rea[2], Camillo Palmieri[1], Elisabetta Caiazzo[3], Carla Cicala[3], Claudio Bellevicine[4], Cristina Falcone[1], Eleonora Vecchio[1], Antonio Pisano[1], Simona Ceglia[1], Selena Mimmi[1], Enrico Iaccino[1], Annamaria de Laurentiis[1], Marilena Pontoriero[1], Valter Agosti[1], Giancarlo Troncone[4], Chiara Mignogna[5], Giuseppe Palma[2], Claudio Arra[2], Massimo Mallardo[6], Franco Maria Buonaguro[2], Giuseppe Scala[1,*] and Ileana Quinto[1,*]

[1]Department of Experimental and Clinical Medicine, University of Catanzaro “Magna Graecia”, Viale Europa, 88100, Catanzaro, Italy

[2]Molecular Biology and Viral Oncogenesis Unit, Department of Experimental Oncology, Istituto Nazionale Tumori "Fondazione Giovanni Pascale", IRCCS, 80131, Naples, Italy.

[3]Department of Pharmacy, University of Naples “Federico II”, Via Domenico Montesano 49, 80131, Naples, Italy.

[4]Department of Public Health, University of Naples “Federico II”, Via Sergio Pansini 5, 80131, Naples, Italy.

[5]Science of Health Department, University of Catanzaro “Magna Graecia”, Italy.

[6]Department of Molecular Medicine and Medical Biotechnology, University of Naples “Federico II”, Via Sergio Pansini 5, 80131, Naples, Italy.

[*]To whom correspondence should be addressed:

Giuseppe Fiume, Phone: +39 0961 3695181; Fax: +39 0961 3694090; Email: fiume@unicz.it

Giuseppe Scala, Phone: +39 0961 3694058; Fax: +39 0961 3694090; Email: scala@unicz.it

Ileana Quinto, Phone: +39 0961 3694059; Fax: +39 0961 3694090; Email: quinto@unicz.it

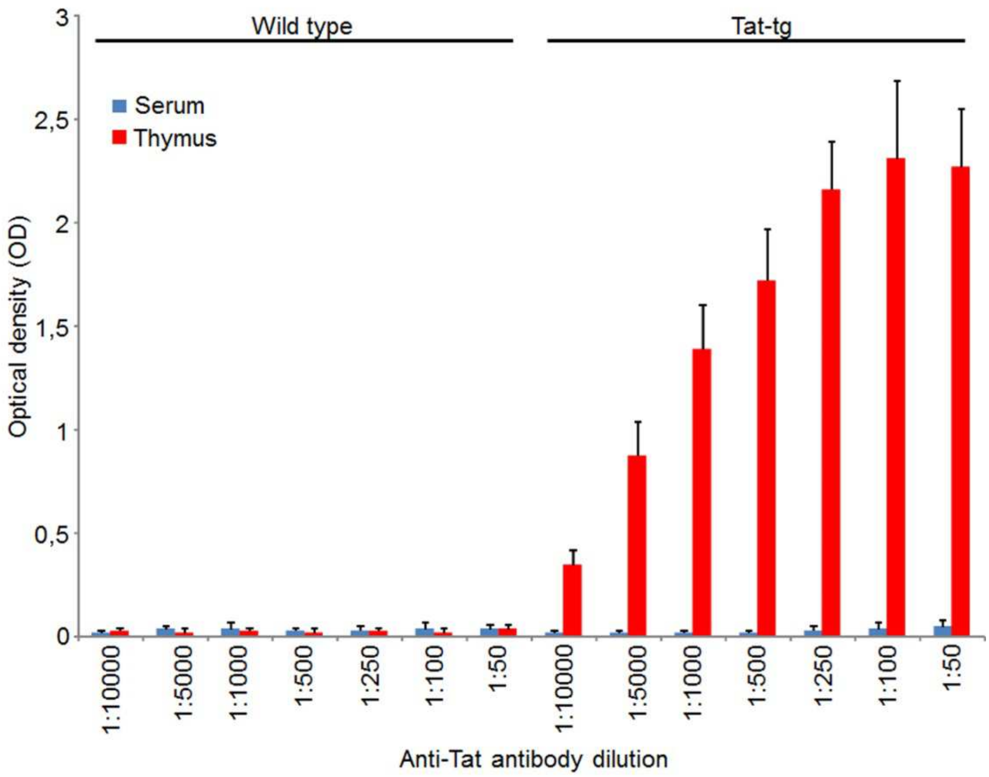

Figure S1. Tat protein expression in the serum and thymus of wild type and Tat-Tg mice. Specific reactivity of serum and thymic proteins (30 µg) of wild type and Tat-Tg mice versus anti-Tat antibody (1 µg/mL) was analysed by ELISA at the indicated dilutions.

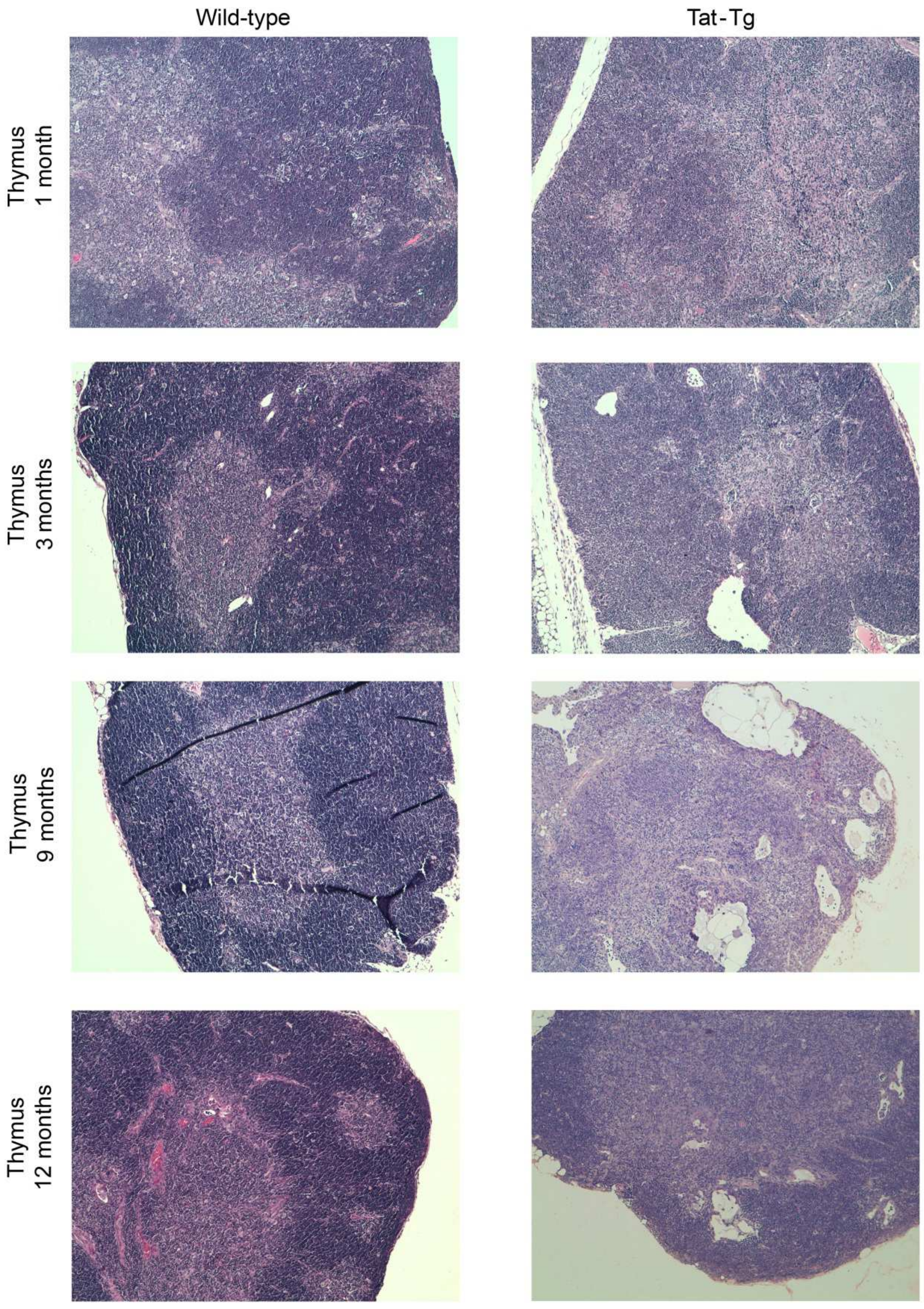

Figure S2. Histological analysis of thymus at different ages.

Thymus sections of wild type and Tat-Tg mice taken at the indicated age were stained by hematoxylin and eosin. Original magnification x 10.

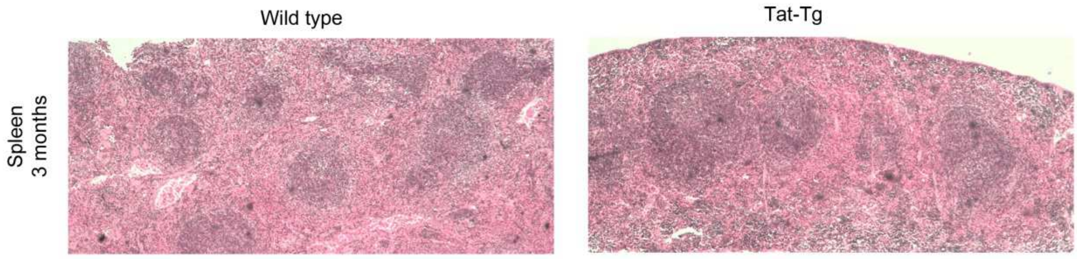

Figure S3. Histological analysis of spleen.

Spleen sections of wild type and Tat-Tg mice were stained by hematoxylin and eosin. Original magnification x 10.

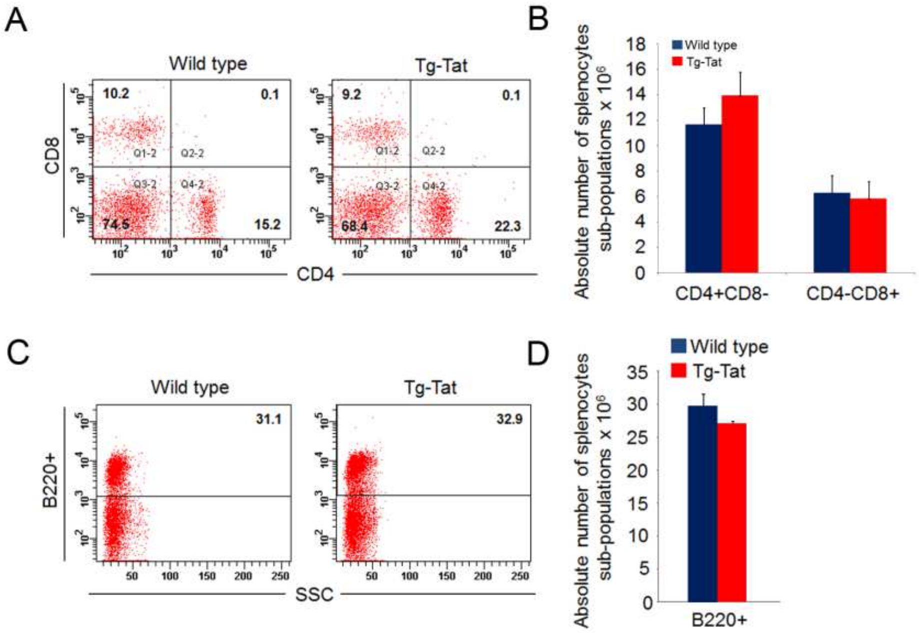

Figure S4. Splenic B and T-lymphocytes subpopulations. (A, B) Percentages and absolute number of SP ($CD4^{+}CD8^{-}$) and SP ($CD4^{-}CD8^{+}$) T-lymphocytes in wild type and Tat-Tg mice. Values (mean ± SE, n = 6) are shown. (**C, D**) Percentages and absolute number of $B220^{+}$ splenocytes of 8 weeks old wild type and Tat-Tg mice. Values (mean ± SE, n = 6) are shown.

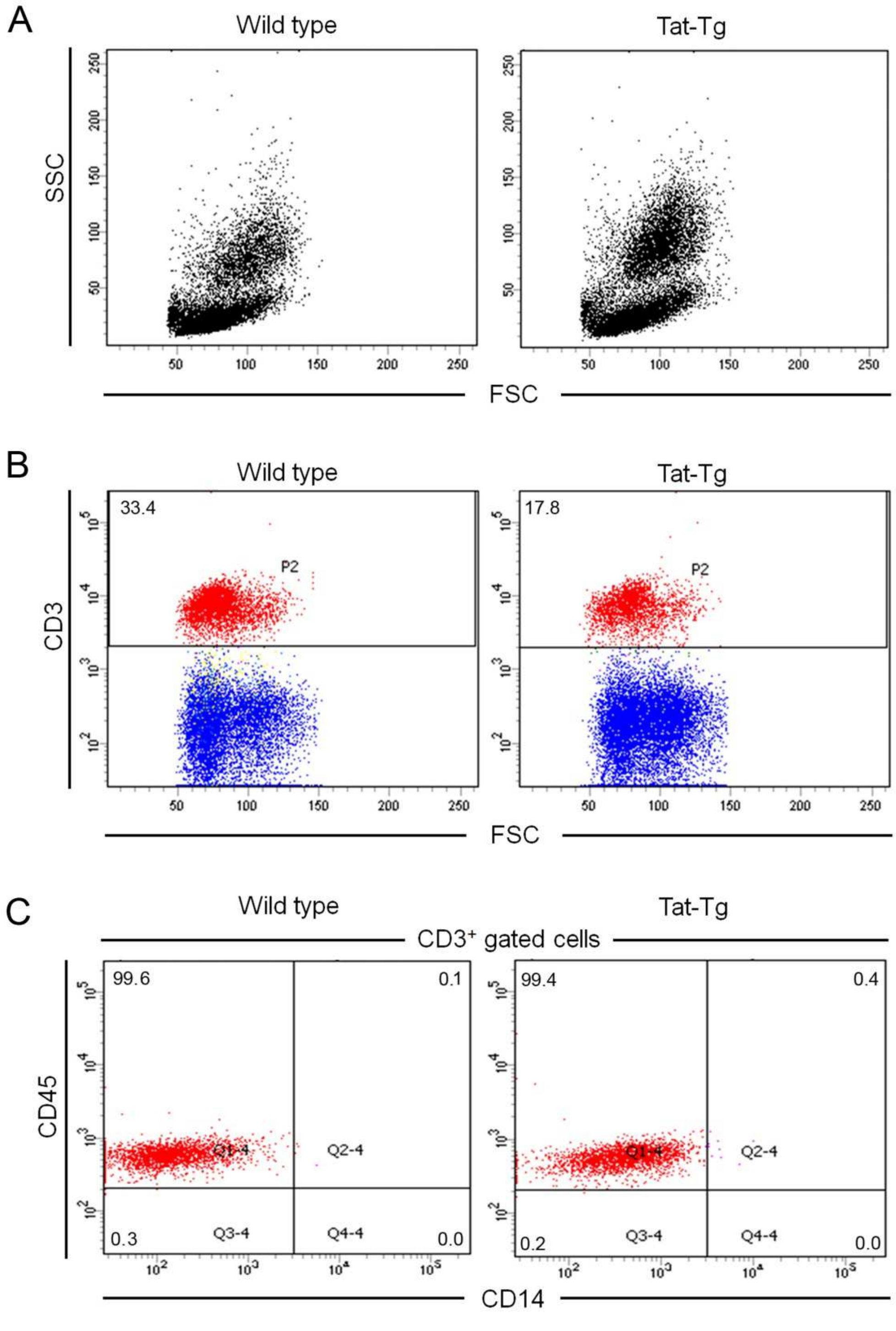

Figure S5. Peripheral blood T-lymphocytes subpopulations. Whole blood cells ($1x10^6$) of 8 weeks old wild type and Tat-Tg mice were stained with anti-CD3-APC, anti-CD45-V500, anti-CD14-V450, anti-CD4-PE and anti-CD8-FITC, and analysed by flow cytometry. (A) Scatter plot of peripheral blood cells of wild type and Tat-Tg mice after red blood cell lysis. (B) Percentages of $CD3^+$ T-lymphocytes of wild type and Tat-Tg mice. (C) Percentages of CD45-CD14-, CD45+CD14+, $CD45^+CD14^-$ and $CD45^-CD14^+$ on $CD3^+$ gated cells are shown.

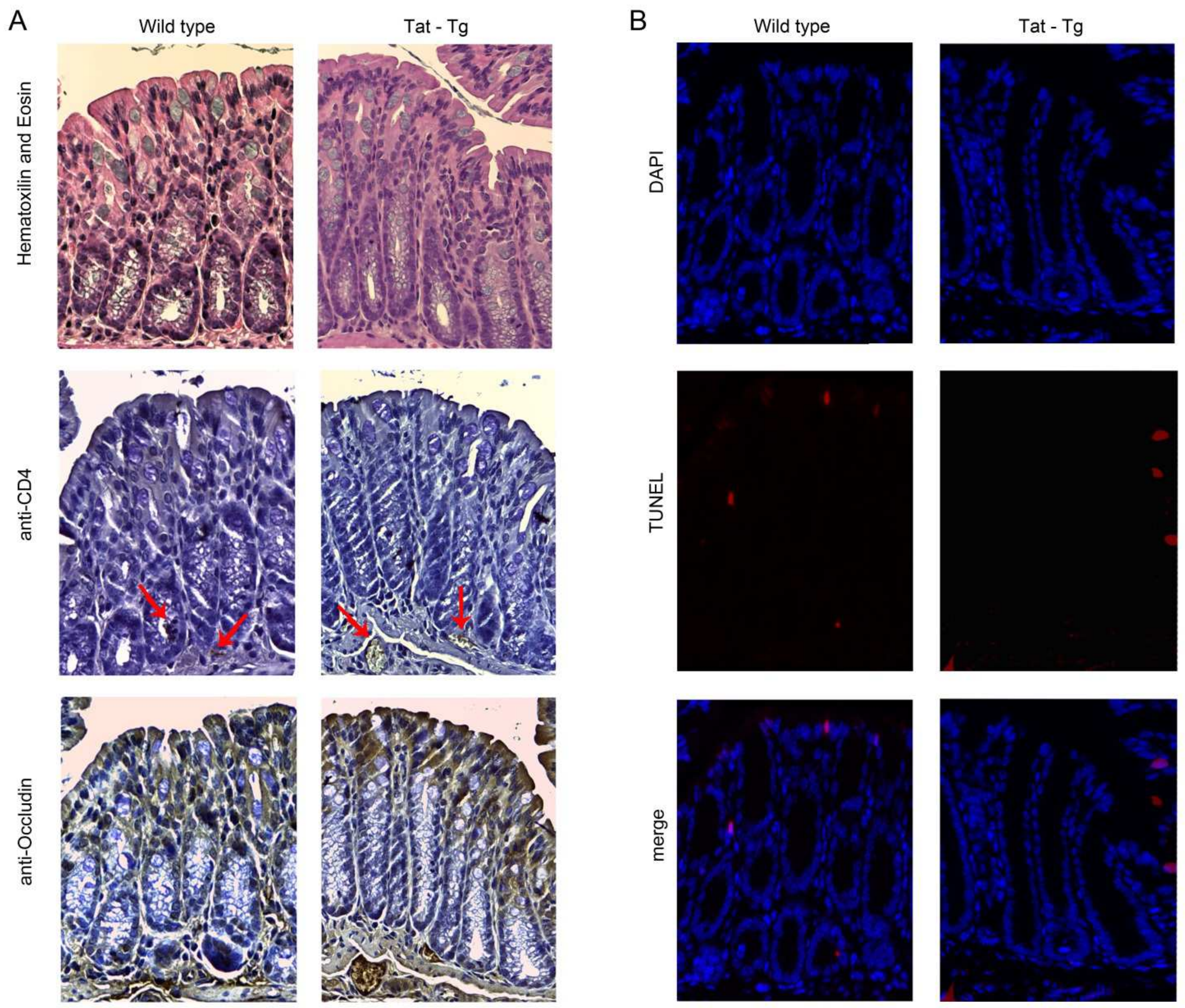

Figure S6. Immunohistochemical analysis of intestinal epithelium

(A) Large bowel mucosa sections of wild type and Tat-Tg mice were stained with hematoxylin and eosin (upper panels); immunohistochemical analysis of large bowel mucosa sections of wild type and Tat-Tg mice using anti-CD4 antibody (middle panels) and anti-Occludin antibody (lower panels). (B) Analysis of the specimens shown in A by TUNEL and DAPI immunofluorescence. Original magnification 40 x.

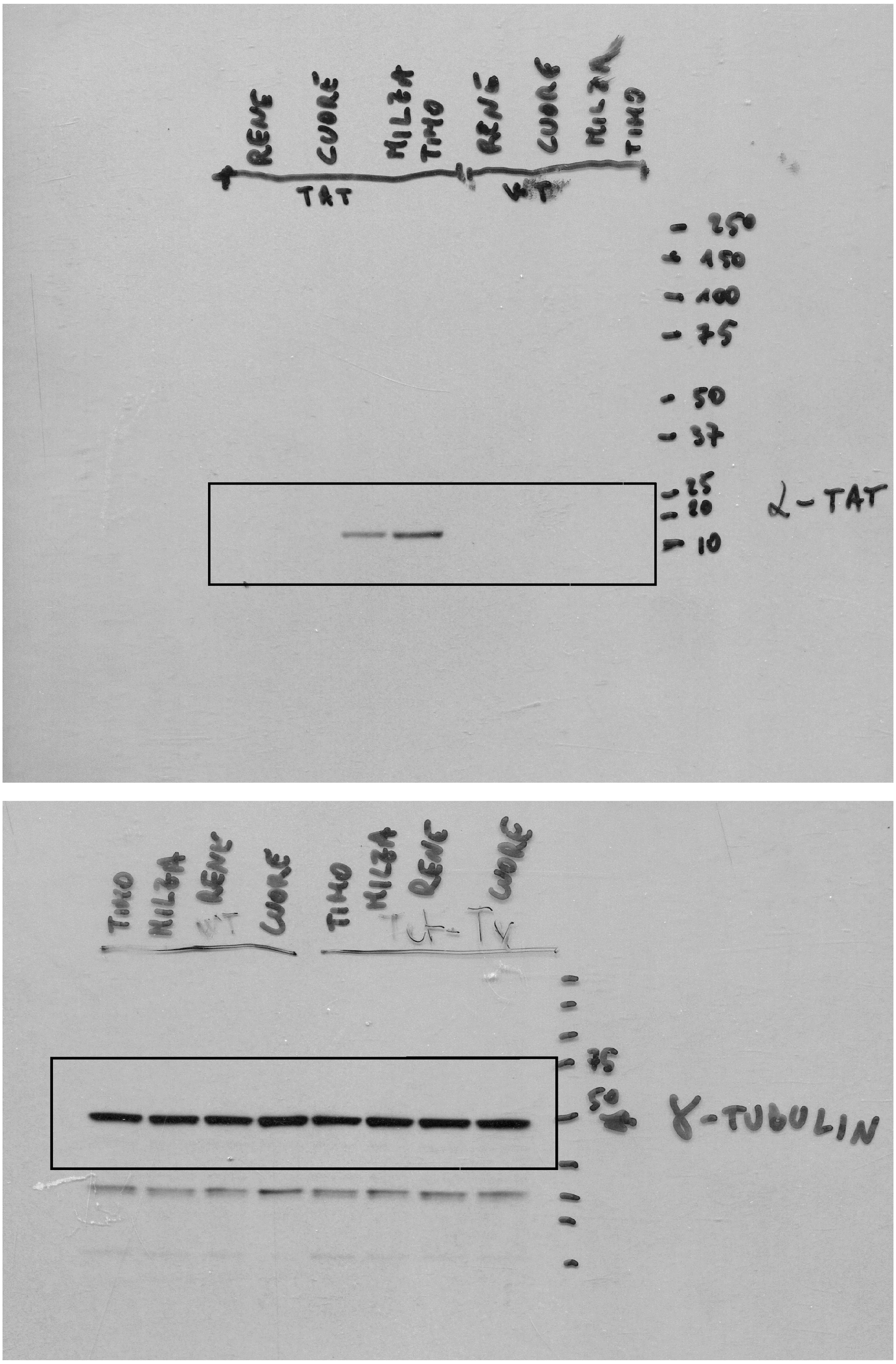

Figure S7. Presentation of original immunoblot shown in Figure 1C.

The cropped parts of immunoblot were indicated with black boxes.

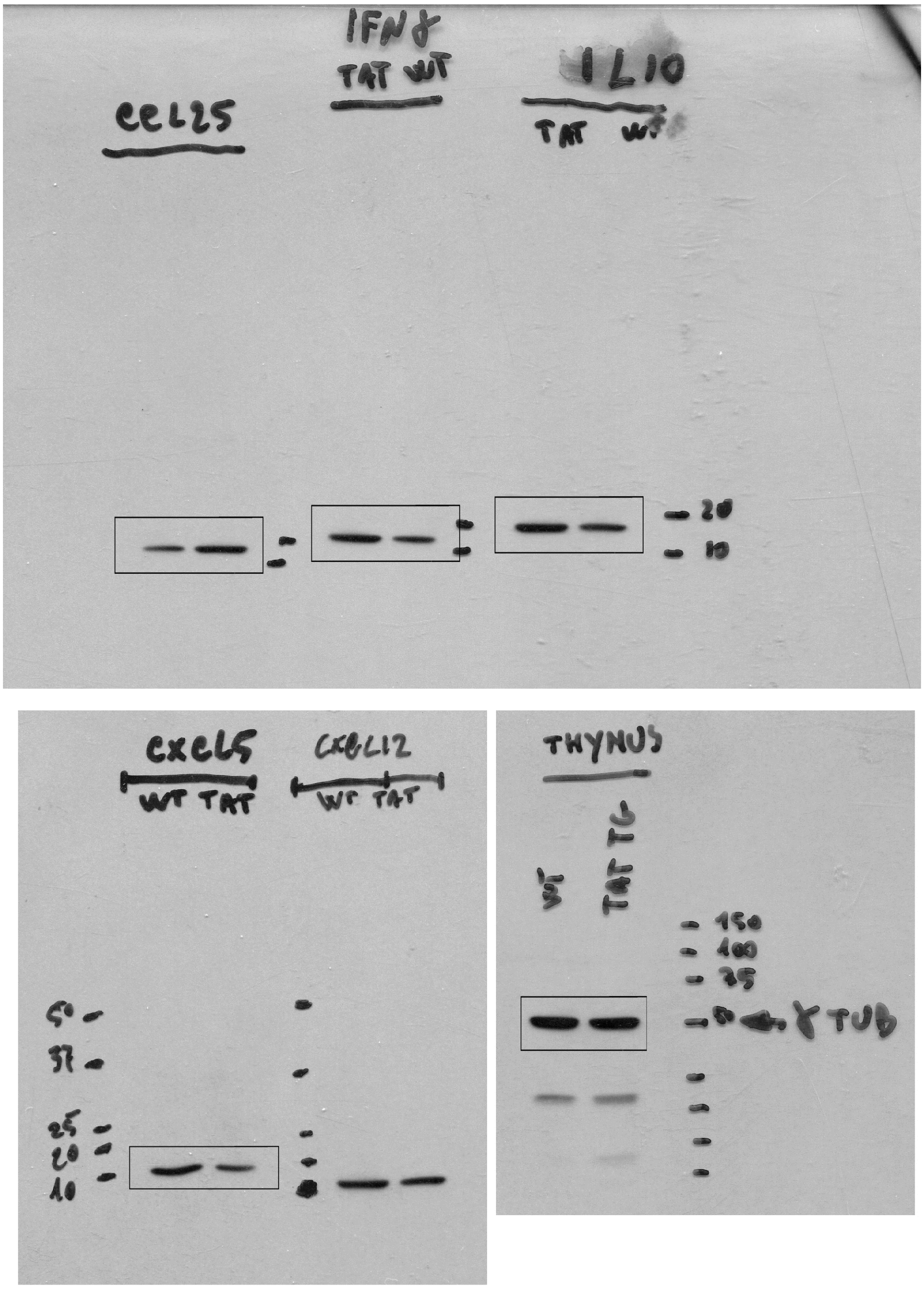

Figure S8. Presentation of original immunoblot shown in Figure 3B.

The cropped parts of immunoblot were indicated with black boxes.

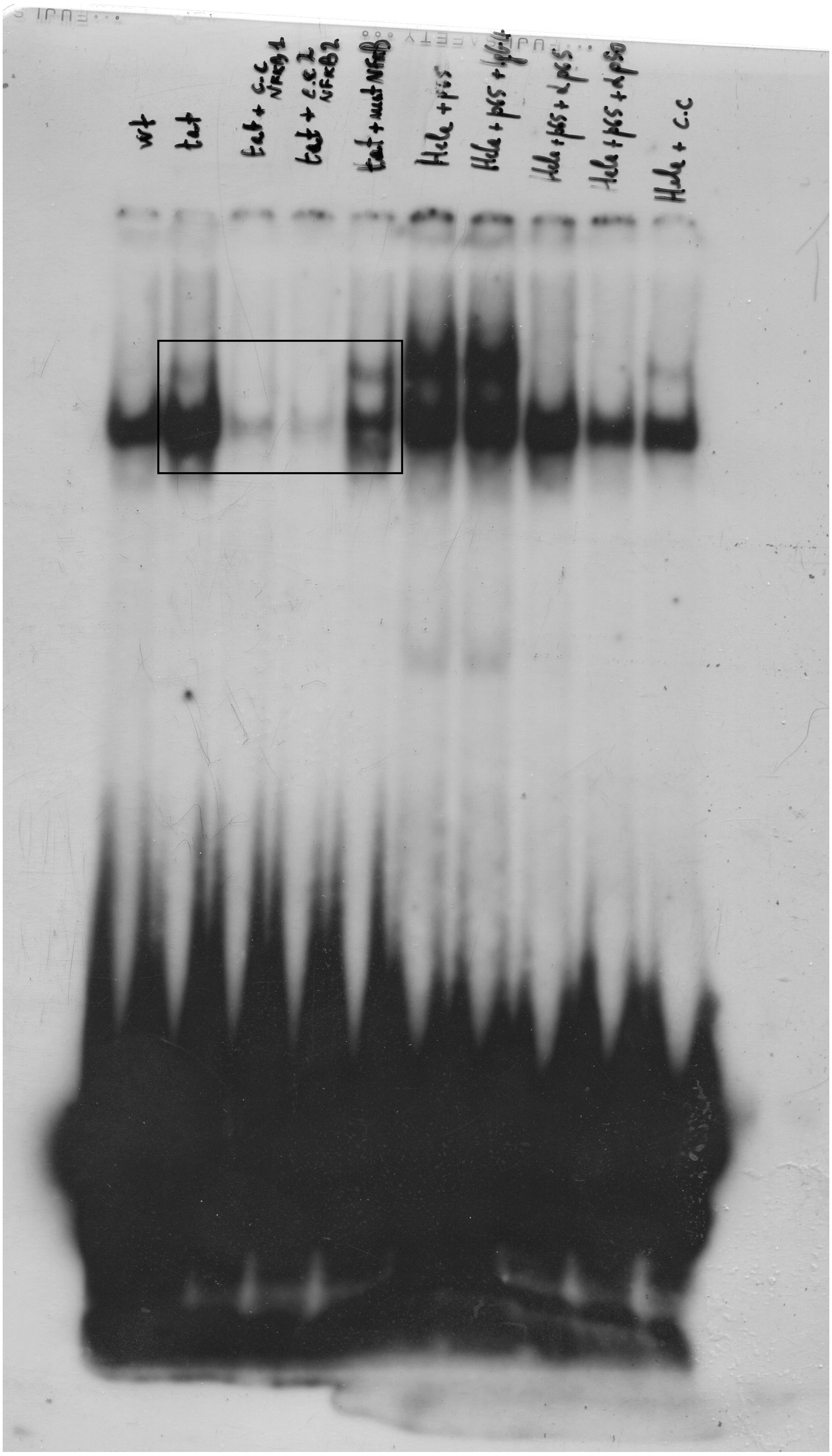

Figure S9. Presentation of original EMSA shown in Figure 3C.

The cropped parts of immunoblot were indicated with black boxes.

Gene Symbol	Wild type mice Thymic Cytokines mRNA Expression (Mean value ± SE)	Tat-Tg mice Thymic Cytokines mRNA Expression (Mean value ± SE)	Tat-Tg versus wild type mice Up/Down-regulated
Abcf1	0.388 ± 0.111	0.348 ± 0.008	-
Bcl6	0.472 ± 0.093	0.394 ± 0.072	-
Cxcr5	0.00728 ± 0	0.00683 ± 0.00142	-
C3	0.040 ± 0.00439	0.0327 ± 0.000747	-
Casp1	0.00747 ± 0.000337	0.00802 ± 0.000497	-
Ccl1	0.00277 ± 0.000866	0.00192 ± 0.000332	-
Ccl11	0.00370 ± 0.000745	0.000207 ± 0.0000690	Down-regulated
Ccl12	0.00455 ± 0.00136	0.00358 ± 0.000752	-
Ccl17	0.0115 ± 0.00515	0.0138 ± 0.00608	-
Ccl19	0.0199 ± 0.0091	0.00115 ± 0.00035	Down-regulated
Ccl2	0.00514 ± 0.0016	0.00595 ± 0.0016	-
Ccl20	0.0161 ± 0.0045	0.00158 ± 0.00013	Down-regulated
Ccl22	0.0570 ± 0.0013	0.0589 ± 0.0017	-
Ccl24	Not detected	Not detected	-
Ccl25	0.630 ± 0.181	0.0305 ± 0.0118	Down-regulated
Ccl3	0.00177 ± 0.000491	0.0158 ± 0.0040	Up-regulated
Ccl4	0.00234 ± 0.0010	0.0285 ± 0.0073	Up-regulated
Ccl5	0.241 ± 0.026	0.191 ± 0.079	-
Ccl6	0.00595 ± 0.0018	0.00629 ± 0.0027	-
Ccl7	0.000575 ± 0.00011	0.00277 ± 0.0010	Up-regulated
Ccl8	0.0375 ± 0.00681	0.00721 ± 0.000293	Down-regulated
Ccl9	0.0322 ± 0.0031	0.0208 ± 0.0034	-
Ccr1	0.00504 ± 0.00023	0.00318 ± 0.00030	-
Ccr2	0.0112 ± 0.0039	0.00889 ± 0.00079	-
Ccr3	0.0291 ± 0.0145	0.0225 ± 0.0033	-
Ccr4	0.581 ± 0.116	0.486 ± 0.146	-
Ccr5	0.0136 ± 0.0077	0.00955 ± 0.0025	-
Ccr6	0.0322 ± 0.0031	0.0236 0.581 ± 0.00054	-
Ccr7	0.307 ± 0	0.283 ± 0.0667	-
Ccr8	0.0299 ± 0.00134	0.0334 ± 0.00978	-
Ccr9	2.51 ± 0.207	2.357 ± 0.471	-
Crp	Not detected	Not detected	-
Cx3cl1	0.00919 ± 0.00264	0.00717 ± 0.00037	-
Cxcl1	Not detected	Not detected	-
Cxcl10	0.0586 ± 0.0182	0.0428 ± 0.0029	-
Cxcl11	0.00300 ± 0.001	0.000201 ± 0.00005	Down-regulated
Cxcl12	0.254 ± 0.0325	0.0898 ± 0.0524	Down-regulated
Cxcl13	0.00231 ± 0.00085	0.00245 ± 0.000178	-
Cxcl15	Not detected	Not detected	-
Pf4	0.00500 ± 0.000915	0.00432 ± 0.00065	-

Cxcl5	0.00165 ± 0.000743444	0.000103 ± 0.00004	Down-regulated
Cxcl9	0.0136 ± 0.0020	0.0127 ± 0.0020	-
Cxcr3	0.0110 ± 0.00162	0.00945 ± 0.00128	-
Ccr10	0.00503 ± 0.00011	0.00575 ± 0.00109	-
Ifng	0.00225 ± 0.00066	0.0111 ± 0.00197	Up-regulated
Il10	0.00148 ± 0.00061	0.0191 ± 0.00908	Up-regulated
Il10ra	0.0297 ± 0.00436	0.0293 ± 0.00209	-
Il10rb	0.0905 ± 0.0260	0.0732 ± 0.0105	-
Il11	Not detected	Not detected	-
Il13	0.00986 ± 0.0037	0.00706 ± 0.0011	-
Il13ra1	0.0120 ± 0.0047	0.00405 ± 0.00030	Down-regulated
Il15	0.0145 ± 0.0033	0.0126 ± 0.00089	-
Il16	0.386 ± 0.13	0.544 ± 0.27	-
Il17b	0.00138 ± 0.00056	0.00151 ± 0.00059	-
Il18	0.0211 ± 0.0060	0.0168 ± 0.0010	-
Il1a	0.00809 ± 0.00087	0.00766 ± 0.0017	-
Il1b	0.00525 ± 0.00077	0.00814 ± 0.0033	-
Il1f6	Not detected	Not detected	-
Il1f8	Not detected	Not detected	-
Il1r1	0.0201 ± 0.00709	0.00331 ± 0.000573	Down-regulated
Il1r2	0.00637 ± 0.00228	0.000969 ± 0.000306	Down-regulated
Il20	Not detected	Not detected	-
Il2rb	0.110 ± 0.0332	0.0744 ± 0.00217	-
Il2rg	2.43 ± 0.295	2.38 ± 0.280	-
Il3	Not detected	Not detected	
Il4	0.00194 ± 0.00081	0.00184 ± 0.00084	-
Il5ra	0.00838 ± 0.00123	0.00594 ± 0.00101	-
Il6ra	0.0288 ± 0.0035	0.0376 ± 0.0058	-
Il6st	0.631 ± 0.028	0.582 ± 0.069	-
Il8rb	0.00852 ± 0.0010	0.00136 ± 0.0010	Down-regulated
Itgam	0.0198 ± 0.0019	0.0163 ± 0.0023	-
Itgb2	0.937 ± 0.067	0.910 ± 0.21	-
Lta	0.00722 ± 0.00087	0.00523 ± 0.00042	-
Ltb	Not detected	Not detected	-
Mif	0.264 ± 0.114	0.387 ± 0.173	-
Scye1	0.312 ± 0.122	0.272 ± 0.108	-
Spp1	0.00740 ± 0.0029	0.00631 ± 0.00046	-
Tgfb1	0.648 ± 0.27	0.562 ± 0.142	-
Tnf	0.0352 ± 0.0060	0.0386 ± 0.0059	-
Tnfrsf1a	0.233 ± 0	0.187 ± 0.074	-
Tnfrsf1b	0.0924 ± 0.010	0.0718 ± 0.0077	-
Cd40lg	0.125 ± 0.102	0.090 ± 0.032	-
Tollip	0.0914 ± 0.025	0.0808 ± 0.0104	-
Xcr1	0.00579 ± 0.00027	0.00577 ± 0.00081	-
Gusb	0.238 ± 0.00558	0.202 ± 0.0437	-
Hprt1	1 ± 0	1 ± 0	-

Table S1. Thymic cytokines and chemokines mRNA expression of 2 months old wild type and Tat-Tg mice. Gene expression levels were calculated relatively to HPRT1 mRNA level. Cytokines and chemokines gene expression was considered as differentially expressed in Tat-Tg mice when the fold increase was above 2 (up-regulated), or less than 0.5 (down-regulated) as compared to wild type, with a statistically significant difference, according to two-tails unpaired Student's *t*-test (p value < 0.05).

Gene Symbol	Wild type mice<br>Serum Cytokines Expression<br>(Mean value ± SE)	Tat-Tg mice<br>Serum Cytokines Expression<br>(Mean value ± SE)	Tat-Tg<br>vs<br>Wild type mice<br>Up/Down-regulated
CXCL13/BCA-1	0.26 ± 0.02	0.61 ± 0.03	Up-regulated
C5/C5α	5.58 ± 0.52	6.96 ± 1.27	-
G-CSF	NOT DETECTED	NOT DETECTED	-
GM-CSF	NOT DETECTED	NOT DETECTED	-
CCL1/TCA-3	NOT DETECTED	NOT DETECTED	-
CCL11	NOT DETECTED	NOT DETECTED	-
sICAM-1(CD54)	8.20 ± 0.57	9.28 ± 1.26	-
INF-γ	0.31 ± 0.02	0.22 ± 0.03	-
IL-1α	0.85 ± 0.12	0.65 ± 0.04	-
IL-1β	NOT DETECTED	NOT DETECTED	-
IL-1RA	1.0694 ± 0.06	1.23 ± 0.65	-
IL-2	NOT DETECTED	NOT DETECTED	-
IL-3	NOT DETECTED	NOT DETECTED	-
IL-4	NOT DETECTED	NOT DETECTED	-
IL-5	NOT DETECTED	NOT DETECTED	-
IL-6	NOT DETECTED	NOT DETECTED	-
IL-7	NOT DETECTED	NOT DETECTED	-
IL-10	NOT DETECTED	NOT DETECTED	-
IL-13	NOT DETECTED	NOT DETECTED	-
IL-12/p70	NOT DETECTED	NOT DETECTED	-
IL-16	NOT DETECTED	NOT DETECTED	-
IL-17	NOT DETECTED	NOT DETECTED	-
IL-23	NOT DETECTED	NOT DETECTED	-
IL-27	NOT DETECTED	NOT DETECTED	-
CXCL10	NOT DETECTED	1.58 ± 0.30	Up-regulated
CXCL11	NOT DETECTED	NOT DETECTED	-
CXCL1	0.30 ± 0.01	0.75 ± 0.16	Up-regulated
M-CSF	3.10 ± 0.45	2.96 ± 0.29	-
CCL2/MCP-1	1.45 ± 0.33	1.82 ± 0.60	-
CCL12	NOT DETECTED	NOT DETECTED	-
CXCL9	NOT DETECTED	0.76 ± 0.17	Up-regulated

MIP-1α	NOT DETECTED	NOT DETECTED	-
MIP-1β	NOT DETECTED	NOT DETECTED	-
CXCL2	NOT DETECTED	NOT DETECTED	-
RANTES	NOT DETECTED	NOT DETECTED	-
SDF-1	0.54 ± 0.07	NOT DETECTED	Down-regulated
TARC	NOT DETECTED	NOT DETECTED	-
TIMP-1	2.46 ± 0.45	5.21 ± 0.58	Up-regulated
TNF-α	0.58 ± 0.08	1.36 ± 0.28	Up-regulated
TREM-1	NOT DETECTED	NOT DETECTED	-

Table S2. Serum cytokines and chemokines expression of wild type and Tat-Tg mice. Proteins were considered as differentially expressed in Tat-Tg mice when the fold increase was above 2 (up-regulated), or less than 0.5 (down-regulated) as compared to wild type, with a statistically significant difference, according to two-tails unpaired Student's *t*-test (*p* value < 0.05).

Gene Symbol	Wild type mice Cytokines Expression within peritoneal fluid (Mean value ± SE)	Tat-Tg mice Cytokines Expression within peritoneal fluid (Mean value ± SE)	Tat-Tg vs wild type mice Up/Down-regulated
CXCL13/BCA-1	0.77 ± 0.03	0.80 ± 0.01	-
C5/C5α	2.34 ± 0.27	3.25 ± 0.38	-
G-CSF	5.37 ± 0.18	5.91 ± 0.22	-
GM-CSF	0.12 ± 0.01	0.51 ± 0.04	Up-regulated
CCL1/TCA-3	0.82 ± 0.06	1.07 ± 0.11	-
CCL11	NOT DETECTED	NOT DETECTED	-
sICAM-1(CD54)	3.36 ± 0.20	3.70 ± 0.14	-
INF-γ	0.24 ± 0.04	0.26 ± 0.01	-
IL-1α	0.48 ± 0.08	1.25 ± 0.11	Up-regulated
IL-1β	NOT DETECTED	NOT DETECTED	-
IL-1RA	1.87 ± 0.16	5.53 ± 0.24	Up-regulated
IL-2	NOT DETECTED	NOT DETECTED	-
IL-3	NOT DETECTED	NOT DETECTED	-
IL-4	0.15 ± 0.02	0.21 ± 0.06	-
IL-5	NOT DETECTED	NOT DETECTED	
IL-6	6.20 ± 0.9	18.16 ± 0.81	Up-regulated
IL-7	0.42 ± 0.11	NOT DETECTED	Down-regulated
IL-10	NOT DETECTED	0.77 ± 0.12	Up-regulated
IL-13	NOT DETECTED	NOT DETECTED	-

IL-12/p70	NOT DETECTED	NOT DETECTED	-
IL-16	NOT DETECTED	0.64 ± 0.07	Up-regulated
IL-17	1.59 ± 0.10	1.87 ± 0.12	-
IL-23	NOT DETECTED	NOT DETECTED	-
IL-27	NOT DETECTED	NOT DETECTED	-
CXCL10	7.62 ± 0.55	15.79 ± 0.36	Up-regulated
CXCL11	0.28 ± 0.01	0.84 ± 0.12	Up-regulated
CXCL1	7.16 ± 0.20	6.60 ± 0.23	-
M-CSF	2.50 ± 0.09	2.58 ± 0.04	-
CCL2/MCP-1	7.71 ± 0.21	5.61 ± 0.18	-
CCL12	0.73 ± 0.02	0.70 ± 0.01	-
CXCL9	9.65 ± 0.79	10.96 ± 0.13	-
MIP-1α	0.26 ± 0.03	0.84 ± 0.05	Up-regulated
MIP-1β	NOT DETECTED	0.40 ± 0.03	Up-regulated
CXCL2	3.90 ± 0.56	13.20 ± 0.88	Up-regulated
RANTES	2.01 ± 0.26	4.29 ± 0.22	Up-regulated
SDF-1	NOT DETECTED	NOT DETECTED	-
TARC	NOT DETECTED	NOT DETECTED	-
TIMP-1	5.58 ± 0.80	10.72 ± 0.44	Up-regulated
TNF-α	0.24 ± 0.04	0.73 ± 0.03	Up-regulated
TREM-1	0.14 ± 0.04	0.13 ± 0.03	-

Table S3. Cytokines and chemokines expression in the peritoneal fluid of wild type and Tat-Tg mice after LPS challenge. Proteins were considered as differentially expressed in Tat-Tg mice when the fold increase was above 2 (up-regulated), or less than 0.5 (down-regulated) as compared to wild type, with a statistically significant difference, according to two-tails unpaired Student's *t*-test (p value < 0.05).
